# Multifaceted Applications of Chitosan in Cancer Drug Delivery and Therapy

**DOI:** 10.3390/md15040096

**Published:** 2017-03-27

**Authors:** Anish Babu, Rajagopal Ramesh

**Affiliations:** 1 Department of Pathology, University of Oklahoma Health Sciences Center, Oklahoma City, OK 73104, USA; anish-babu@ouhsc.edu; 2 Stephenson Cancer Center, University of Oklahoma Health Sciences Center, Oklahoma City, OK 73104, USA; 3 Graduate Program in Biomedical Sciences, University of Oklahoma Health Sciences Center, Oklahoma City, OK 73104, USA

**Keywords:** chitosan, gene delivery, drug delivery, adjuvant, cancer, nanoparticle

## Abstract

Chitosan is a versatile polysaccharide of biological origin. Due to the biocompatible and biodegradable nature of chitosan, it is intensively utilized in biomedical applications in scaffold engineering as an absorption enhancer, and for bioactive and controlled drug release. In cancer therapy, chitosan has multifaceted applications, such as assisting in gene delivery and chemotherapeutic delivery, and as an immunoadjuvant for vaccines. The present review highlights the recent applications of chitosan and chitosan derivatives in cancer therapy.

## 1. Introduction

**Figure 1:**
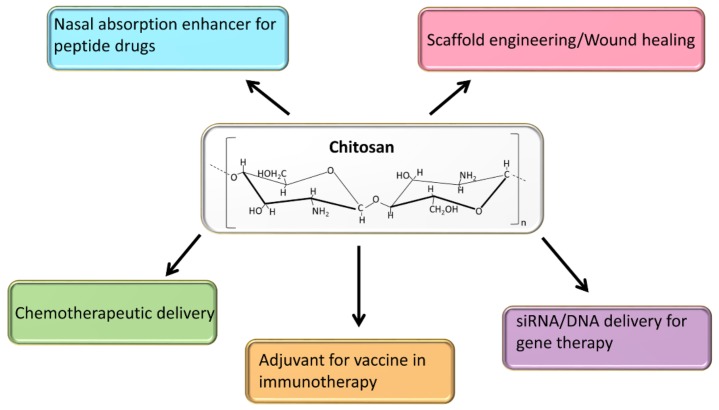
Diagram showing the various applications of chitosan in healthcare and cancer therapy. Abbreviations: siRNA (small interfering siRNA).

Marine products have been in the forefront of natural materials used in therapeutic applications against human diseases [1]. The marine biopolymer chitin, which is isolated from crustaceans and is the second most abundant polymer in nature, has recently received increased attention for healthcare applications [2]. Chitin transforms into chitosan by partial deacetylation under strong alkaline conditions. Chitosan is composed of (1 → 4)-2-acetamido-2-deoxy-β-d-glucan (*N*-acetyl d-glucosamine) and (1 → 4)-2-amino-2-deoxy-β-d-glucan (d-glucosamine) units, and has long been used in health care materials, such as nasal absorption enhancers of peptide drugs [3], and in 3D or 2D scaffold preparations for wound healing [4]. In addition, chitosan has been widely used as a promising non-viral delivery vector for biomacromolecules and low molecular weight drugs [5]. Figure 1 shows the major applications of chitosan in healthcare and cancer therapy. Chitosan exhibits high biocompatibility and biodegradability, attractive properties for the development of a safe and active drug delivery tool [5,6]. Chitosan is cationic in nature and its solubility in water is poor but it is soluble in low pH solutions. Different derivatives of chitosan have been made to overcome this limitation for controlled drug delivery purposes [6,7]. The cationic charge of chitosan has been utilized for ionic gelation methods using materials with strong anionic charge for nanoparticle preparation [8]. Additionally, this cationic nature has been harnessed for electrostatic interaction with nucleic acids, and chitosan has been used as a gene delivery carrier for cancer therapy. Another important application of chitosan as a potential immune-adjuvant for cancer vaccines has been realized recently [9]. The present review summarizes some of the significant recent developments in which chitosan is used as nanoparticle carrier for gene therapeutics, chemotherapeutic drugs, and in immune-adjuvant therapy for cancer.

## 2. Chitosan as Gene Delivery Vehicle for Cancer Therapy

In gene therapy, a gene of interest that has been implicated in cancer pathology is altered or manipulated by delivering exogenous nucleic acid material into the tumor cells or the milieu [10]. However, when nucleic acid therapeutics are systemically administered, they encounter many hurdles across their circulation to reach the target tissue in the human body that might reduce their therapeutic potential. These hurdles include short plasma half-life due to enzymatic degradation and rapid bio-clearance of nucleic acid therapeutics from the circulation. In vivo circulating nucleic acid therapeutics also face cellular entry limitations, such as charge-based repulsion from cell membranes and poor endosomal escape. To overcome these barriers, delivery vehicles are required for nucleic acids [11,12].

Viral or non-viral vectors are major systems used for gene delivery applications. Viral vectors are excellent transfection agents, however mutagen and carcinogen properties of many viral vectors limits their use in cancer gene therapy [13]. As an alternative to viral-vector nanotechnology, non-viral vectors have made remarkable advances in recent years [14]. Non-viral vectors for gene delivery include liposomes, polymer-based carriers and nanoparticles of various kinds. Among them, liposome is a highly-investigated gene carrier because of its high transfection efficiency and ease of preparation. However, poor encapsulation efficiency, short shelf-life, non-specific toxicity and low in vivo stability are major limitations of liposomes [15,16,17]. Though PEGylation (PEG: poly-ethylene glycol) appears to improve the circulation time of liposomes, the accelerated blood clearance (ABC) phenomenon resulting from repeated liposome administration enhanced its bio-clearance from the body [18]. As alternative gene carriers, cationic polymers were extensively used for gene delivery due to their improved transfection efficiency, high gene encapsulation and in vivo stability [19]. The presence of numerous free amine groups in cationic polymers such as polyethylene imine (PEI), chitosan (CS), poly-l-lysine (PLL) and polyamidoamine (PAMAM) effectively condense oligonucleotides or DNA. The high cationic charge density in these polymers allow for enhanced intracellular trafficking of nanoparticles via endosomal disruption, however this imparts undesired cellular toxicity [20]. Interestingly, chitosan is an exception in that it exhibits no apparent toxicity as gene delivery vehicle. Chitosan has excellent physicochemical properties that appear to be favorable for nucleic acid delivery by overcoming the systemic barriers of gene delivery. Chitosan readily forms complexes, microspheres or nanoparticles upon electrostatic interaction with nuclei acids [21]. Because of these promising characteristics, chitosan has been increasingly studied as a gene delivery system in cancer therapy.

### 2.1. Influencing Factors in Chitosan-Based Gene Delivery

The charge-to-charge ratio between chitosan and DNA is a critical factor for successful electrostatic binding of DNA or siRNA to chitosan [21]. The nitrogen-to phosphate (N/P) ratio, i.e., the ratio between the positively charged nitrogen of free amines in chitosan to negatively charged phosphates in nucleic acids potentially influences the efficiency of the chitosan polymer to condense and protect the DNA or siRNA. Reports suggests that a low N/P ratio would affect the stability of chitosan-DNA complexes, whereas extremely high N/P ratios results in low transfection efficiency [22,23].

Molecular weight and de-acetylation degrees are other important factors that determine the stability of chitosan-nucleic acid complexes. The molecular weight of chitosan has a broad range from low molecular weight (LMW, <100 KDa) to medium molecular weight (MMW, <300 KDa) and high molecular weight (HMW, >300 KDa). The ability of chitosan to transfect functionally active siRNA into cells is strongly dependent on the molecular weight of chitosan, among other factors [24]. Huang et al. (2005) reported that a 213-KDa chitosan formulation showed superior uptake and transfection efficiency with plasmid DNA (pDNA) in cancer cells compared with a LMW chitosan formulation [25]. In their report, the LMW chitosan was less capable of condensing and protecting DNA, and could not retain DNA upon dilution [25]. However, there are reports of superior cell uptake and gene delivery using LMW chitosan in cancer cells compared to higher molecular weight chitosan used in the formulations [23,26]. The enhanced cell uptake and transfection efficiency can be attributed to relatively low binding of LMW chitosan to DNA, allowing for easy dissociation compared to higher molecular weight chitosan. Reports also suggest that LMW chitosan formed small nanoparticles and showed significant transfection efficiency for DNA or siRNA polyplexes, when chitosan nanoparticles were modified with targeting ligands [27,28] or with other polymers [29]. When complexed with 25 and 50 kDa of chitosan, siRNA showed <220-nm size and good gene silencing effect in HeLa cells [30]. When LMW chitosan conjugated with another cationic polymer protamine was used for gene delivery at physiological pH, the transfection efficiency and gene expression in host cells were significantly improved [29]. This complex reportedly had low toxicity both in vitro and in vivo. These studies indicate that molecular weight has a great influence in chitosan’s biological and physicochemical properties. However, literature lacks a unanimous opinion about appropriate chitosan molecular weight to be chosen for the best possible transfection efficiency. Nevertheless, the above studies suggest that the chitosan used for gene transfection should have intermediate degree of stability and appropriate molecular weight, which appears to be better achieved with LMW chitosan, that provides a balance between DNA protection ability and intracellular release [21,31].

Along with molecular weight, the degree of deacetylation in chitosan is important in determining its efficiency for stable complex formation with DNA or siRNA. Most of the chitosan used in gene delivery applications has a high degree of deacetylation (DDA), since high DDA corresponds to more free amines and increased positive charge for efficient DNA binding. Moreover, studies suggest that the binding efficiency of nucleic acids decreased with decreasing DDA in chitosan, resulting in incomplete complex formation [23]. This is because chitosan with low DDA has fewer primary amine groups that are freely available for electrostatic interaction with negatively charged nucleic acids. Based on reports, it is recommended that DDA % should be above 65% in chitosan for efficient complex formation with DNA [32]. The particle size also has been shown to decrease upon increased DDA % of chitosan when complexed with DNA [25].

The pH of the transfection medium must also be considered, since the protonation of amine groups in chitosan requires an acidic pH range (5.0–6.0). This low acidic pH increases the DNA binding efficiency of chitosan and thereby enhances transfection efficiency [27]. The presence of serum is an important factor that determines the stability of a cationic gene delivery system [27]. Interestingly, compared with other cationic polymers, chitosan-based DNA transfection is improved in the presence of serum [33]. Sato et al. (2001) studied the transfection efficiency of pDNA/chitosan complexes in the presence of serum (0%–50%). Their data showed that the 20% serum conditions resulted in highest transfection efficiency, whereas 50% serum in the medium produced the lowest transfection efficiency [26].

The difference in size and charge density of DNA and siRNA influence the complex formation with chitosan of same length. Due to the bigger size and good charge density of plasmid DNA, it has the ability for strong electrostatic binding compared to small-sized siRNA (19–25 bp) [21]. Taken together, to develop a successful gene delivery system using chitosan, all of the above-mentioned parameters should be considered carefully. Apart from the physicochemical characteristics of chitosan-gene complexes, the cell type may influence the transfection efficiency. Therefore, when handling a difficult-to-transfect cell-line, the chitosan-gene complexes should be tailor-made. This includes the use of chitosan derivatives and the addition of other polymers that favor the best possible transfection efficiency in the specific cell line.

### 2.2. Formulation Methods

**Figure 2:**
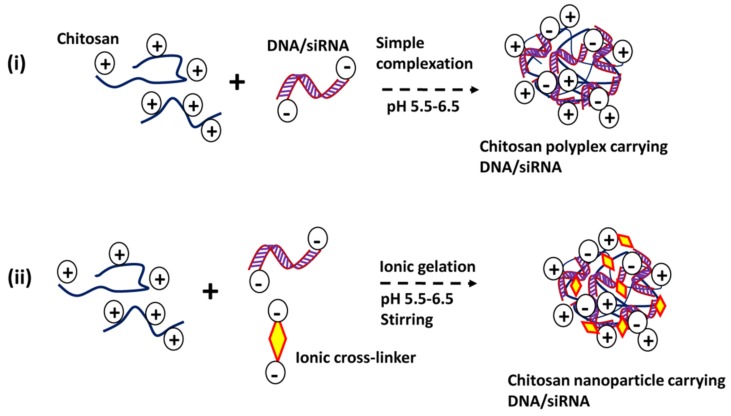
Common preparation methods of chitosan nanocarrier for DNA/siRNA delivery. (**a**) simple complexation; (**b**) ionic gelation.

There are two commonly used formulation methods for chitosan-based gene delivery systems: (a) simple complexation and (b) ionic gelation (Figure 2). Simple complexation between the chitosan polymer and siRNA or DNA involves electrostatic interactions between cationic chitosan and anionic nucleic acids [34,35,36]. At proper N/P ratios, chitosan forms complexes in micro- or nano-sized particles. Formation of smaller particles requires optimization of molecular weight, DDA, pH, and sometimes external force, like mild stirring, for proper condensation and complex formation. Ionic gelation is a common method to prepare crosslinked nanoparticles. In ionic gelation, the siRNA or DNA is entrapped, rather than fully depending on electrostatic interactions [8,37]. Crosslinkers that are oppositely and strongly negatively charged to chitosans, such as tripolyphosphate, thiamine pyrophosphate, and hyaluronic acid, are used in the ionic gelation process. While crosslinking enhances the stability of the nanoparticles, it slows down the release of entrapped nucleic acids. Nevertheless, this crosslinking strategy may be useful in gene delivery that requires slow and sustained release of nucleic acids over time.

### 2.3. Chitosan Derivatives in Gene Delivery

Poor water solubility in physiological pH is a limitation of chitosan in gene delivery applications. Since chitosan requires the protonation of its free amines for effective complexation with siRNA or DNA, which is possible only at acidic pH, transfection in physiological pH may result in early dissociation of siRNA into the medium without achieving effective cellular transfection [38]. Another issue is the slow release of nucleic acid materials from chitosan, possibly affecting transfection efficiency. Therefore, chitosan derivatives were synthesized by chemical modifications of chitosan structure or by grafting polymers with distinct properties to overcome water insolubility and poor gene delivery efficiency. Quaternization is a commonly used method to modify chitosan by alkylation of tertiary amines by different methods for improved gene transfection [39,40]. A recent example used a quaternary ammonium salt crystal called *N*-2-hydroxypropyl trimethyl ammonium chloride chitosan (HACC) for gene delivery in human cells [41]. The HACC particles were not cytotoxic, and HACC/pDNA complexes showed comparable transfection efficiency to liposome/pDNA complexes, indicative of their potential as a novel tool for gene delivery. Chitosan hydroxybenzotriazole (chitosan-HOBT) is another derivative of chitosan known for its safe and efficient siRNA delivery capacity [42]. Chitosan-HOBT could condense siRNA, formed stable complexes, and exhibited good gene silencing efficiency. However, its full gene therapeutic potential in cancer cells is yet to be realized. Another study used dendronized chitosan derivative prepared by modification of 6-azido-6-deoxy-chitosan with propargyl focal point poly(amidoamine) dendron [43]. Compared with PEI non-viral vector, these novel dendronized chitosan/DNA complexes showed enhanced gene transfection efficiency in human kidney and nasopharyngeal carcinoma cells. In a different derivatization approach, hybrid-type chitosan (MixNCH) was synthesized using 2-chloroethylamine hydrochloride and N, *N*-dimethyl-2-chloroethylamine hydrochloride, for gene delivery to cancer cells [44]. MixNCH nanoparticles showed good physicochemical characteristics for gene delivery, transfected HepG2 cancer cells, and effectively inhibited cell proliferation.

Trimethyl chitosan (TMC) is one of the intensively studied quarternized derivatives of chitosan in gene delivery applications [45,46,47,48,49]. One of the important advantages of trimethylation is that chitosan’s solubility can be increased in physiological pH. Compared to chitosan polyplexes, trimethyl chitosan nanoparticles strongly reduces the aggregation tendency and pH dependency of nucleic acid complexation [45]. Studies in NIH/3T3 (mouse embryonic fibroblasts) cells showed a huge increase in transfection efficiency of pDNA using TMC nanoparticles compared to chitosan polyplexes [45]. Moreover, the same study showed that PEG grafting onto TMC enhanced the particle stability, decreased particle size in physiological pH and reduced the toxicity showed by unmodified TMC. Finally, PEG-TMC nanoparticles enhanced the transfection efficiency of pDNA by 10-fold compared to unmodified TMC.

Conjugating targeting moieties to TMC enhanced the gene delivery efficiency according to a report by Zheng et al. [46]. The TMC nanoparticles efficiently condensed pDNA and the presence of folate on its surface allowed its target-specific delivery of pDNA in SKOV3 (human ovarian adenocarcinoma) and KB (HeLa contaminant, carcinoma) cells which overexpresses folate receptor. The drug carrying ability of TMC nanoparticles has also been harnessed in drug gene co-delivery towards cancer cells. In a recent study, the triple negative MDA-MB-231 (human breast adenocarcinoma) cell line was successfully transfected by high mobility group protein 2 (HMGA-2) siRNA with simultaneous delivery of chemotherapeutic doxorubicin using a TMC nanoparticle system [49]. The anti-cancer effect of doxorubicin has been enhanced by conjunctional delivery of siRNA that silenced HMGA-2 gene expression. All these studies point towards the importance of various factors such as particle size, stability, toxicity, targeting ability and the modifications required for TMC-based gene delivery systems to achieve successful gene transfection.

### 2.4. PEG Modification of Chitosan in Gene Delivery

**Figure 3:**
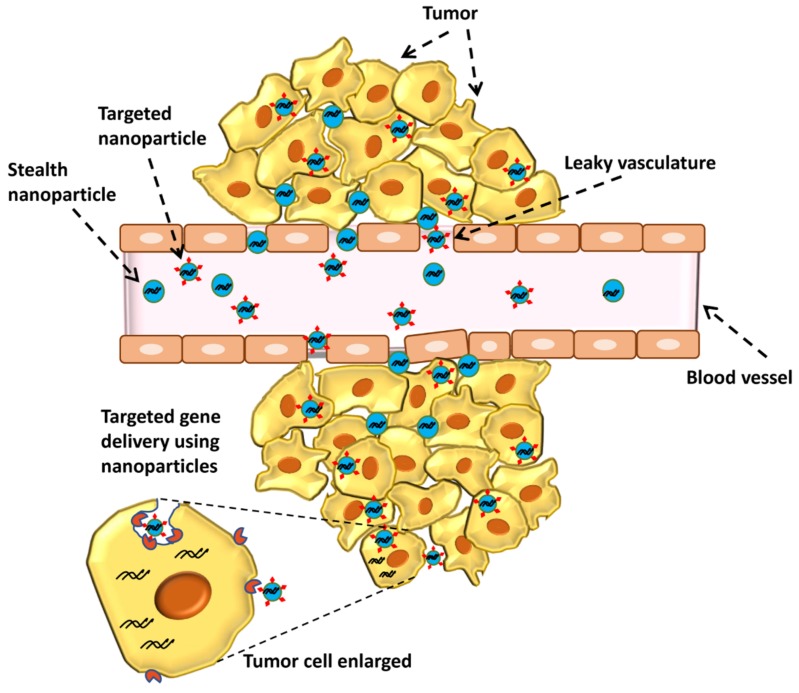
Gene delivery to tumors using PEGylated (stealth) nanoparticles or by using receptor targeted nanoparticles. Nanoparticle (stealth or targeted) enter tumor area via leaky vasculature, while targeted nanoparticles specifically enter tumor cells via receptor mediated pathway (see enlarged portion of the figure). Gene therapeutics are then released into the cytoplasm escaping from the endo-lysosomes. PEG: poly-ethylene glycol.

To make chitosan more water soluble and enhance its blood-circulation time, conjugation of poly-ethylene glycol (PEG) polymer with chitosan is a common approach [50]. The PEGylated nanoparticle is generally known as “stealth” nanoparticle. PEG is a neutral polymer that increases the hydrophilicity of chitosan and delays the reticulo-endothelial system clearance while in the circulation. This improves the chances of the chitosan-gene delivery system to passively accumulate in tumor areas by enhanced permeation and retention effect (EPR) in a time-dependent manner. However, the EPR effect applies to only those nanoparticles with particle sizes less than 200 nm in most cases. Moreover, PEGylation may reduce the charge-based affinity of cationic chitosan towards net negatively charged cell membranes and affect the cellular delivery of gene therapeutics. This issue has been addressed by attaching targeting ligands or stimuli responsive polymers to nanoparticles for receptor target delivery of nucleic acids to tumor cells. While PEG improves the circulation half-life of nanoparticles, conjugation of targeting ligands enhances cell-specific delivery of gene therapeutics [30] (Figure 3). PEG also serves as the linker molecule for nanoparticle modification with targeting ligands. Chan et al. (2007) developed a chitosan gene delivery system with PEG-folate modification for targeted delivery to folic acid receptor-overexpressing tumor cells [51]. This chitosan nanoparticle system carrying DNA not only improved the water solubility upon PEG addition, but also showed low cytotoxicity towards normal HEK 293 (Human embryonic kidney cells 293) cells. A recent study demonstrated the use of transferrin (Tf)-functionalized chitosan nanoparticles, where PEG was used to conjugate Tf onto chitosan [52]. Thus, PEG modification is an important step in designing water-soluble, long-circulating, and target-specific nanoparticles.

Altogether, chitosan is a promising gene delivery system for in vitro and in vivo applications, however requires several formulation parameters to be optimized. Structure modification or incorporation of other polymers is an effective way to enhance the potential of chitosan by improving the in vivo stability, target specificity and desirable intracellular release of gene therapeutics. Some recent examples of chitosan-based gene delivery an application are described in the Table 1.

## 3. Chitosan Nanoparticles in Chemotherapeutic Delivery

Nano-drug delivery systems using chitosan offer many advantages. These systems minimize drug clearance in the circulation, control release of drug, reduce drug cytotoxicity, and increase therapeutic index. Moreover, the biodegradability and biocompatibility have made chitosan a suitable material for chemo-drug delivery in cancer therapy. Chitosan is mucoadhesive, and its cationic nature allows for enhanced affinity towards mucous membrane, thereby assisting trans-mucosal drug delivery. These properties of chitosan would be useful in intra-nasal and intrapulmonary delivery of chemotherapeutics for cancers especially of the nasopharyngeal and lung tissues.

### 3.1. Delivery of Hydrophilic Chemotherapeutics

Chitosan nanoparticles can be used to deliver both hydrophilic drugs [61,62], and hydrophobic drugs [63,64]. The presence of many free amine groups can be easily functionalized for conjugation of chemotherapeutic drugs. For example, in a recent study, water-soluble drug doxorubicin (DOX) was conjugated to chitosan using a succinic anhydride spacer [62]. The succinic anhydride could react with the amine of DOX and functionalize to become carboxylic. This carboxylic acid of DOX was then conjugated with chitosan’s free amine groups using carbodiimide chemistry. The chitosan-DOX was then self-assembled to form nanoparticles in aqueous solution under stirring at room temperature. However, the introduction of more DOX reduced the conjugation efficiency to chitosan. The Her2+ (human epidermal growth factor receptor 2+) targeting monoclonal antibody, trastuzumab was also conjugated to chitosan-DOX nanoparticles via thiolation of lysine residues (by reacting with primary amines) and subsequent linking of the resulted thiols to chitosan. The trastuzumab conjugated chitosan-DOX nanoparticles showed target specificity towards Her2+ cancer cells, resulting in enhanced uptake compared to chitosan-DOX and free drug. Also, trastuzumab conjugated chitosan-DOX nanoparticles could efficiently discriminate between Her2+ and Her2− cells, demonstrating its potential for active targeted drug delivery.

In another strategy, a chitosan-pluronic micelle was designed and fabricated for the encapsulation of water-soluble DOX [65]. They grafted Pluronic^®^ F127 polymer into chitosan and fabricated a co-polymer micelle that can encapsulate DOX with high drug loading capacity with a particle size of 50 nm. The chitosan-pluronic micelle carrying DOX (DOX-NP) showed better in vitro therapeutic activity than free DOX in MCF7 breast cancer cell lines.

### 3.2. Delivery of Hydrophobic Chemotherapeutics

For the delivery of poorly water-soluble drugs, chitosan derivatives have been synthesized with suitable characteristics that can support hydrophobic drugs. Paclitaxel, a hydrophobic chemotherapeutic, showed enhanced activity when encapsulated in a glyceryl monooleate-chitosan core-shell nanoparticle prepared using an emulsification-evaporation technique [66]. Strikingly, a 1000-fold reduction in paclitaxel IC_50_ (Inhibitory Concentration 50) was observed with this core-shell nanosystem in MDA-MB-231 human breast cancer cells. This huge reduction in IC_50_ value would reduce the cytotoxicity of paclitaxel towards normal cells. In a different study, Kim et al. (2006) introduced an amphiphilic derivative of chitosan for paclitaxel delivery [63]. They combined glycol chitosan and 5β-cholanic acid to produce nanoparticles (Glycol chitosan hydrophobically modified with 5beta-cholanic acid or HGC nanoparticles). The drug loading achieved for paclitaxel was 80% in HGC nanoparticles. The cytotoxicity of HGC nanoparticles were negligible compared to conventional Cremophor EL formulation used for paclitaxel administration. Further, when administered in mice tumor model, the tumor regression ability of paclitaxel delivered using HGC nanoparticles was comparable to Cremophor EL at 20 mg/kg dose, whereas a higher concentration of paclitaxel (50 mg/kg) in HGC nanoparticles caused complete regression of tumors in four out of six treated mice. Their study clearly indicated a superior anticancer effect of HGC nanoparticle formulation paclitaxel compared to Cremophor EL formulation. Later, the same group studied cisplatin (CDDP) loaded-HGC nanoparticles for their physicochemical properties intended for anti-cancer therapy [67]. CDDP, a low water soluble drug (up to 1 mg/mL) was encapsulated in hydrophobic cores of HGC nanoparticles and showed sustained drug release. In vivo delivery of CDDP-HGC nanoparticles accumulated in solid tumors in a mouse model via the EPR effect. Finally, they showed promising antitumor efficiency of CDDP-HGC nanoparticles in tumor-bearing mice.

Chitosan-copolymer nanoparticles are also used to encapsulate hydrophobic anti-cancer drug 5-flurouracil (5-FU), as reported by Rajan et al. [68]. They prepared a hyaluronidase-5-fluoruracil (5-FU)-loaded chitosan-PEG-gelatin polymer nanocomposite using the ionic gelation technique. A short-time incubation (3–12 h) of hyaluronidase-5-fluoruracil (5-FU)-loaded chitosan formulations showed less toxicity than chemotherapeutic 5-FU. Hyaluronic acid conjugation with biopolymers imparted targeting capability for the drug delivery vehicle towards cancer cells. The physicochemical characteristics such as particle size, homogenous distribution, morphology, drug loading capacity and low toxicity of these chitosan-based nanocomposite formulations are promising for the drug delivery system in anti-cancer studies.

Recently, Cavalli et al. (2014) formulated chitosan nanospheres with 5-fluorouracil using a combination of coacervation and emulsion droplet coalescence methods [69]. The resulting 5-FU-loaded chitosan nanospheres were not only able to reduce the proliferation of HT29 (Human colorectal adenocarcinoma) and PC-3 (Human prostate cancer-3) tumor cell lines in a time- and concentration-dependent manner but also inhibited their adhesion to human umbilical vein endothelial cells (HUVEC). These examples suggest that chitosan-based nanoparticles have the potential to deliver a wide range of drugs with different physicochemical properties. Table 2 shows some recent examples of chitosan or chitosan-based nanoparticles in chemotherapeutic drugs of hydrophilic, hydrophobic or amphiphilic properties for cancer therapy.

### 3.3. Targeted Delivery of Chemotherapeutics Using Chitosan-Based Nanoparticles

**Figure 4:**
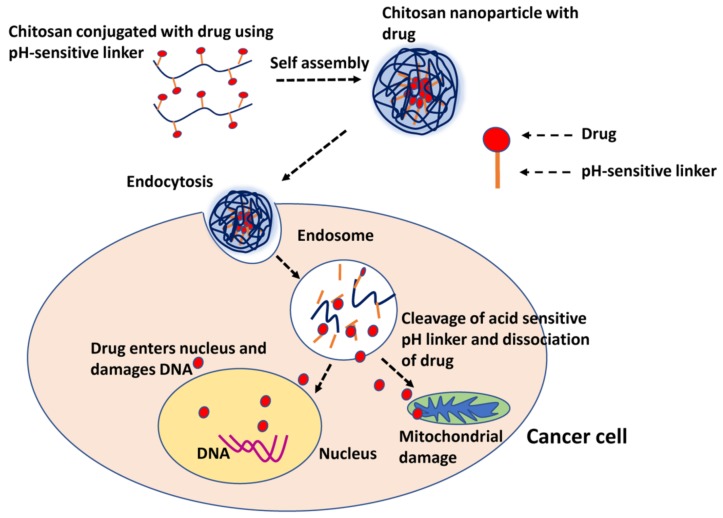
Acid responsive drug delivery using chitosan nanoparticles. Chitosan is linked to drug molecules with a pH-sensitive linker. After endocytotic uptake of nanoparticles, the pH-sensitive linker is dissolved (bond breakage) in the acidic pH of the endosomes, resulting in the release of conjugated drug into the cytoplasm. The drug is then transported to the nucleus or mitochondria and causes DNA damage and apoptosis.

Conjugation of tumor-specific ligands onto chitosan nanoparticles has been developed for active targeting [78]. Many surface receptors specifically overexpressed in cancer cells are exploited for receptor-targeted delivery of chemotherapeutics using chitosan nanoparticles. Specific interaction between targeting ligands in nanoparticles and cell surface receptors results in receptor-mediated endocytosis nanoparticles. In cells, the internalized drug-loaded chitosan nanoparticles escape from endo-lysosomal compartments and accumulate in cytoplasm, where the nanoparticles release the drug payload over time. Transferrin, epidermal growth factor receptor (EGFR), folate receptor, CD44 (known as HCAM or homing cell adhesion molecule) receptor, integrins, and low density lipoprotein receptors are commonly exploited for targeted drug delivery in cancer cells [79]. The expression levels of these receptors in each cancer type varies; therefore, it is important to know the cell type and receptor expression levels before formulating targeted drug delivery systems. When conjugated with drug via pH-cleavable bonds, chitosan nanoparticles undergo dissociation of the assembly within the acidic pH of endo-lysosomes and release the drug into the cytoplasm [80]. Figure 4 shows the diagrammatic representation of stimuli responsive drug delivery of chitosan-based nanoparticles with acid-cleavable bonds conferred by a pH-sensitive linker.

## 4. Chitosan in Cancer Immunotherapy

Vaccines require adjuvants for enhancing the immune response. Aluminum hydroxide, lipopolysaccharide derivative monophosphoryl lipid A, antimicrobial peptide, and TLR9 (Toll like receptor 9) combinations were among the adjuvants commonly used with vaccines. However, due to possible side effects, scientists worldwide are in search of safe and potential adjuvants for vaccine development, especially in cancer therapy.

Polysaccharides from plant, animal, and fungal sources have emerged as possible adjuvants for cancer vaccines [81]. Among these, chitosan has the potential to become an ideal vaccine adjuvant due to its safety, biocompatibility, cationic nature, and its ability to be used as an antigen carrier [82]. For more than two decades, the immunostimulatory activity of chitosan has been known. However, its potential as a safe and non-toxic adjuvant in cancer vaccine development has only recently been realized [9,83]. Recent studies explored the adjuvant properties of chitosan in vaccines against cancer and infectious diseases [9,83,84,85]. The bioadhesive property of chitosan aids in its cell-uptake, leading to strong systemic and mucosal immune responses.

The striking feature of chitosan is that it can enhance both humoral and cell-mediated immune responses [86]. Chitosan showed comparable potency to incomplete Freund’s adjuvant, and showed immune activity superior to that of the traditional immunoadjuvant, aluminum hydroxide (Imject Alum) [87]. Chitosan retains the peptide antigen in the administration site for a longer time, allowing antigen to be presented for efficient immune activity. Zaharoff et al. (2007) reported that more than 60% of antigen is retained in the subcutaneous site of injection, even after 7 days [87]. This strategy may reduce the booster doses of vaccine to be used for enhanced immune response.

The mechanism of immune-adjuvant activity of chitosan has recently been elucidated. Chitosan induces immune activity via the NLRP3 (NLR Family Pyrin Domain Containing 3) inflammasome in phagocytic cells and promotes IL-1β (Interleukin 1β) secretion [88]. It is also reported that chitosan induces mitochondrial DNA-mediated cGAS-STING (Cyclic GMP-AMP synthase- Stimulator of Interferon Genes) pathway activation, resulting in the secretion of IFN (Interferon) type I. IFN type I in turn stimulates the maturation of dendritic cells, resulting in antigen presentation, followed by a Th1 (Type 1 T helper) immune response [89]. Chitosan is also known to elicit a balanced Th1/Th2 immune response [90].

**Figure 5:**
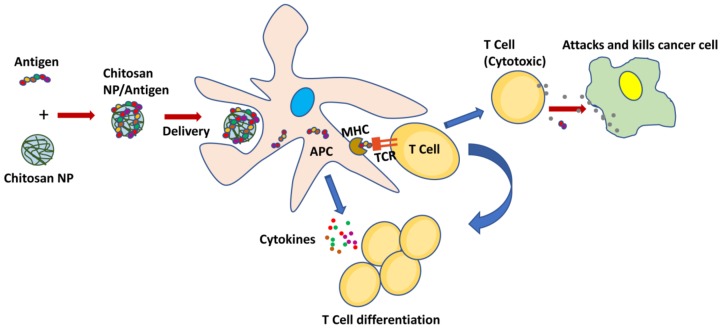
Chitosan nanoparticles act as carriers and enhance the immunostimulatory activity of protein antigen for its presentation by antigen-presenting cells (APC). The stimulated cytotoxic T cells attack and kill cancer cells, whereas cytokines released by APC activate T cell differentiation and expansion. MHC: major histocompatibility complex; TCR: T cell receptor; Chitosan-NP: Chitosan-nanoparticle

A simplified schematic of chitosan’s adjuvant activity when delivering cancer vaccine is depicted in Figure 5. Zaharoff et al. (2010) reported the use of chitosan as adjuvant for IL-12 therapy in colorectal (MC32a) and pancreatic (Panc02) solid tumors in mice [85]. Upon intratumoral injection, chitosan prolonged the retention of IL-12 in the injection site and resulted in tumor regression in more than 80% of mice. The resultant systemic tumor immunity was able to prevent tumor recurrence. As a result of chitosan/IL-12 therapy, CD8^+^ (Cluster of differentiation 8+) cells and NK (Natural killer) cells were revealed as the predominant immune cells involved in the regression of aggressive murine tumors. The same group also demonstrated the efficacy of chitosan/IL-12 adjuvant therapy in superficial bladder cancer treatment [91].

In a different study, Heffernan and colleagues (2011) explored the chitosan/IL-12 adjuvant system in stimulating protein vaccine immune responses [92]. Protein-based vaccines have potential for cancer immunotherapy; however, their poor immunostimulatory effect is a limitation. The immunoadjuvant consisted of a viscous chitosan solution and Il-12 cytokine; when injected along with ovalbumin (OVA; model protein antigen), this treatment elicited increased antigen-specific CD4^+^ and CD8^+^ T-cell responses. Further, the chitosan/IL-2 adjuvant system enhanced IgG2a and IgG2b (Imunoglobulin G2a and b) antibody responses to OVA. Another study reported that chitosan nanoparticles enhanced the Th1 and Th2 immune responses induced by OVA in mice [90]. Chitosan nanoparticles improved not only Th1 (IL-2 and IFN-γ) and Th2 (Il-10) cytokine levels but also increased the killing activity of NK cells. Therefore, chitosan may be a safe and promising immune-adjuvant for cancer vaccine, by promoting both humoral and cellular immune responses.

Since chitosan comprise of a large group of glucosamine polymers, its proper standardization is warranted, although challenging, for the development of a successful vaccine adjuvant. Key characteristics, such as chitosan’s molecular weight, degree of deacetylation, viscosity, and endotoxin levels [93], should be considered when testing chitosan for adjuvant applications. It would be helpful to refer Vasiliev’s (2015) step-by-step approaches in the proper evaluation and standardization of chitosan for use as vaccine-adjuvants [94].

## 5. Conclusions

Chitosan, the natural biodegradable and non-toxic polymer, holds promise as a suitable material for biomedical applications. There are multifaceted applications of chitosan in cancer therapy, including gene delivery, chemotherapeutic delivery, and immunotherapy. Although chitosan-based drug delivery systems and gene delivery vectors are not yet approved by the FDA (Food and Drug Administration), great progress in cancer therapy research is being made. Physico-chemical characteristics, such as its cationic nature, molecular weight, DDA, and pH of transfection medium are major factors that influence the gene delivery efficacy of chitosan nanoparticles. The genetic material, i.e., siRNA or DNA, and cell type also contribute to the efficiency of transfection using chitosan vectors.

However, chitosan’s low water solubility is a major limitation for gene and drug delivery applications. To improve the water solubility, new functional groups or addition of neutral polymers like PEG have been commonly employed. PEG addition also has the advantages of prolonged in vivo circulation and reduced bio-clearance of chitosan nanoparticles. Alone, chitosan has difficulty encapsulating hydrophilic drugs; therefore, conjugation strategies are employed to achieve high drug loading. Derivatization of chitosan with hydrophobic molecules or polymers has enhanced the ability of chitosan to encapsulate hydrophobic drugs.

Targeting of ligands or antibodies is frequently used to improve the target specificity of chitosan in gene or drug delivery applications for cancer. Intracellular delivery of therapeutics can be improved by modification of chitosan with stimuli-responsive polymers or moieties. Apart from these, the immune-adjuvant properties of chitosan are highly promising. Chitosan is known to induce both humoral and cellular immune responses and enhance the immune-stimulatory activity of cancer vaccines. However, the choice of chitosan polymer for immunotherapy is still a challenge, since chitosan is a generically used name for all forms of de-acetylated chitins with versatile properties. Importantly, the molecular weight, DDA, and endotoxin levels of chitosan should be considered in immunoadjuvant applications of chitosan. Worldwide, researchers are engaged in the development of cancer vaccines. It is hoped that chitosan’s promising characteristics as an immunoadjuvant will be advantageous for its future application in cancer vaccines. Overall, chitosan’s multifaceted characteristics show the potential of this marine biopolymer in cancer therapy applications.

## Figures and Tables

**Table 1:** Recent examples of gene delivery systems based on chitosan for cancer therapy. PEG: poly-ethylene glycol.

Chitosan or Chitosan-Associated Nanoparticles	Gene Material/Molecular Target	Cancer/Cell Type	Special Features of the Study/Formulation	Reference
Low molecular weight (LMW) chitosan/2-acrylamido-2-methylpropane sulphonic acid	Model pDNA/Luc (plasmid DNA/Luciferase)	A549 (lung adenocarcinoma), HeLa (cervical carcinoma) and HepG2 (hepatocellular carcinoma)	Incorporation of 2-acrylamido-2-methylpropane sulphonic acid made chitosan water soluble. Higher transfection efficiency in cancer cells and mouse model.	[7]
Alginic acid-coated chitosan nanoparticles	Legumain pDNA	Murine 4T1 (mouse mammary tumor cell line)	Used as oral delivery system for DNA vaccine. Legumain pDNA delivery improved autoimmune response to breast cancer in mice.	[53]
Glycol-chitosan nanoparticles	MDR1 (Multi drug resistant 1)-siRNA	MCF-7 (Human breast adenocarcinoma; Adriamycin resistant, ADR)	Nanoparticles accumulated in MCF7/ADR tumors and downregulated P-gp expression. Chemo-siRNA combination therapy significantly inhibited tumor growth without systemic toxicity in mice.	[54]
Polyethylene glycol-chitosan	Survivin-siRNA	Murine 4T1 (mouse mammary tumor cell line)	The PEG–Chitosan nanoparticles carrying siRNA were efficiently taken up by cancer cells and induced antitumor activity in xenografts.	[55]
Biotinylated chitosan-graft-polyethyleneimine	antiEGFR (Epidermal growth factor receptor)-siRNA	Hela (cervical carcinoma), OVCAR-3 (Human ovarian adenocarcinoma)	The biotinylated chitosan-graft-polyethyleneimine was less cytotoxic than polyethyleneimine. Efficient cell uptake and epidermal growth factor siRNA delivery was possible in cancer cells.	[56]
Folate-targeted chitosan polymeric nanoparticles	METHFR (Methylenetetrahydrofolate Reductase) shRNA (coloaded with 5-FU)	SGC-7901 (Human gastric carcinoma)	Folate-targeted chitosan polymeric nanoparticles (CPNs) could reverse drug-resistant SGC-7901 cells by co-delivery of METHFR shRNA and 5-fluorouracil (5-FU). Folate-targeted CPN system showed significantly enhanced therapeutic efficacy compared to non-targeted CPN.	[57]
Polyethyleneimine/poly(allylamine)-citraconic anhydride/gold nanoparticle (PEI/PAH-Cit/AuNP)-chitosan nanoparticle	MDR1 (Multi drug resistant 1) siRNA	MCF-7 (Human breast adenocarcinoma; drug-resistant)	Gold nanoparticle reduced and stabilized by chitosan was coated by charge-reversible polymer PAH-cit and PEI by layer-by-layer deposition. This charge-reversible core/shell nanosystem were effective in protecting, cell uptake and endosomal escape of siRNA; facilitated safe siRNA delivery and gene silencing in cancer cells.	[58]
Chitosan	Plasmid IL-12 (Interleukin-12)	WEHI-164 (Human fibrosarcoma)	Chitosan formed polyplex with IL-12 plasmid. Treatment with IL-12 resulted in significant tumor regression in mouse fibrosarcoma model.	[59]
Chitosan/Polylactic-acid nanoparticle	Plasmid Beta-5/siP62 (P62 or Sequestosome 1 siRNA)	2008S, 2008/C13 (Human ovarian carcinoma; drug-resistant)	Chitosan-coated polylactic acid nanoparticles were co-loaded with siRNA/pDNA and chemotherapeutic. Drug resistant ovarian cancer cells were sensitized to cisplatin by simultaneous delivery P62 siRNA, Proteasome beta-5 plasmid and cisplatin.	[60]

**Table 2:** Examples of chemotherapeutic delivery using chitosan or chitosan based nanoparticles.

Solubility Property	Chemotherapeutic	Nanoparticle	Special Features/Application	Cancer Model/Cell Lines	Reference
Hydrophilic	Doxorubicin	Chitosan diacetate and chitosan triacetate nanoparticles	Sustained release of anticancer drugs Increased oral bioavailability of doxorubicin in animal model	MCF-7 and Caco-II tumor cell lines	[70]
		Cholesterol-modified glycol chitosan (CHGC) self-aggregated nanoparticles	High drug loading (9.36%) and enhanced drug release in low pH range Prolonged circulation in plasma	S180 murine cancer	[71]
		Self-assembled chitosan-doxorubin conjugate (CS-DOX) nanoparticles	Trastuzumab decoration enhanced the uptake of CS-DOX nanoparticles in Her2+ cancer cells compared with nontargeted CS-DOX nanoparticles	MCF7 (breast cancer) and SKOV3 (ovarian cancer) cell lines	[62]
		CD44 targeted-doxorubicin-encapsulated polymeric nanoparticle surface decorated with chitosan	Drug release in acidic tumor environment Nanoparticle delivery Increased cytotoxicity to cancer-stem cells by six times compared to free doxorubicin	3D mammary tumor spheroids	[72]
Hydrophobic	Taxanes	Paclitaxel-loaded chitosan nanoparticles	Nanoparticle exhibited sustained release pattern of paclitaxel Low hemolytic toxicity observed for nanoparticles compared to free drug Nanoparticle demonstrated enhanced antitumor activity in vitro compared to naïve drug	MDA-MB-231 breast cancer cell lines	[73]
		Ionically cross-linked docetaxel loaded chitosan nanoparticles	Nanoparticles exhibited 78%–92% drug encapsulation efficiency Nanoparticle delivery enhanced cytotoxicity of docetaxel compared to free drug	MDA-MB-231 breast cancer cell lines	[74]
		Paclitaxel-loaded *N*-octyl-*O*-sulfate chitosan micelles	*N*-octyl-*O*-sulfate chitosan inhibited p-glycoprotein overcoming multi-drug resistance Paclitaxel- *N*-octyl-*O*-sulfate chitosan micelles showed superior blood persistence, tumor accumulation, and therapeutic efficacy in tumor bearing mice	Human hepatocellular liver carcinoma (HepG2) cells and the multidrug resistance HepG2 (HepG2-P) cells	[75]
Sparingly-water soluble	Platinum drugs	Folic acid-conjugated chitosan-coated poly(d-l-lactide-co-glycolide) (PLGA) nanoparticles (FPCC)	Presence of protective chitosan layer controlled the overall release rate of carboplatin FPCC displayed higher cell uptake and reduced IC_50_ (Inhibitory concentration 50) values of carboplatin compared to non-targeted nanoparticles	Hela cervical cancer cells	[76]
		Cisplatin-loaded cholanic acid-modified glycol chitosan nanoparticles	Drug loading was 80% Cisplatin-loaded nanoparticles showed prolonged blood circulation and accumulated in tumor by utilizing enhanced permeation and retention effect (EPR) effect Nanoparticles delivery showed higher anti-tumor efficacy and lower toxicity compared to free cisplatin	MDA-MB231 human breast tumor	[67]
		Cisplatin loaded- chitosan-nanolayered solid lipid nanoparticles (CChSLN)	Nanoparticle exhibited excellent biocompatibility IC_50_ value of cisplatin was lowered by CChSLN delivery CChSLN enhanced apoptosis in cancer cells compared to free cisplatin	HeLa cervical carcinoma	[77]
